# Secondary analysis of hand-offs in internal medicine using the I-PASS mnemonic

**DOI:** 10.1186/s12909-024-05880-7

**Published:** 2024-09-27

**Authors:** Aurélie Huber, Belinda Moyano, Katherine Blondon

**Affiliations:** 1 Faculty of Medicine, University of Geneva, Geneva, Switzerland; 2 University Hospitals of Geneva, Geneva, Switzerland

**Keywords:** Hand-off, Sign-out, I-PASS, Relevance, Completeness

## Abstract

**Background:**

Miscommunications account for up to 80% of preventable medical errors. Mnemonics like I-PASS (Illness severity, Patient summary, Actions list, Situation awareness, Synthesis) have demonstrated a positive impact on reducing error rates. Currently, physicians at our hospital do not follow a specific structure during hand-offs. We aimed to compare current hand-offs without prior training to a gold standard and the I-PASS tool in terms of content and sequence.

**Methods:**

This study is a secondary analysis of data collected during a simulation study of a Friday evening hand-off to the night resident at University Hospitals of Geneva. Thirty physicians received a hand-off of four patients and managed two other patients through nursing pages at the start of the night shift, generating six sign-outs each, totaling 177 sign-outs. A focus group of three senior doctors defined the gold standard (GS) by consensus on the essential content of each sign-out. The analysis focused on the rates of relevance (ratio of information considered relevant by the GS) and completeness (proportion of transmitted elements out of all expected elements of the GS), and the distribution and sequence of the first four I-PASS categories.

**Results:**

Relevance and completeness rates were 37.2% ± 0.07 and 51.9% ± 0.1, respectively, with no significant difference between residents and supervisors. There was a positive correlation between total hand-off time and relevance (residents: R^2^ = 0.62; supervisors: R^2^ = 0.67) and completeness (residents: R^2^ = 0.32; supervisors: R^2^ = 0.56). The distribution of I-PASS categories was highly skewed in both the GS (I = 2%, *P* = 72%, A = 17%, S = 9%) and participants (I = 6%, *P* = 73%, A = 14%, S = 7%), with significant differences in categories A (*p* = 0.046) and I (*p* ≤ 0.001). Sequences of I-PASS categories generally followed a P-A-S-I pattern. The first S category was frequently absent, and only one participant began by announcing the case severity as suggested by I-PASS.

**Conclusion:**

We identified gaps between current medical sign-outs in our institution's general internal medicine division and the I-PASS structure. We recommend implementing the I-PASS mnemonic, emphasizing the "I" category at the start and the "S" category to anticipate and prevent complications. Future studies should assess the impact of this recommendation, adapt the mnemonic elements to the context, and introduce specific hand-off training for senior medical students.

**Supplementary Information:**

The online version contains supplementary material available at 10.1186/s12909-024-05880-7.

## Background

A hand-off can be described as a transfer of patient information and accountability between two health professionals [1], and is required to ensure the continuity of care. Prior studies about hand-offs have shown that errors in hand-offs can potentially endanger patient safety [2–4].

A particularly critical situation is the takeover of the night shift. The physician on night duty will be responsible for a higher number of patients, most of whom she is unfamiliar with; there will also be less supervision by senior doctors during the night.

Hand-offs are particularly prone to error. Indeed, it has been shown that ineffective medical hand-offs are responsible for 80% of serious medical errors and two-thirds of sentinel events [1] ("unplanned events causing death or serious physical injury or psychological harm, or related risks" [5, 6]. This risk could, however, be avoided [7] or reduced by structuring and training medical hand-offs using mnemonics [8, 9]. Structures help to avoid forgetting important features. Standardization of these communications would reduce the number of medical errors and thus could have a positive impact on healthcare costs by reducing 1) length of stay; 2) diagnostic delay; and 3) repeated testing [9].

In 2011, Amy Starmer and her team developed the I-PASS [10] mnemonic tool:

- I) Illness severity
- P) Patient summary (summary statement, events leading up to admissions, hospital course, ongoing assessment, plan)
- A) Actions list
- S) Situation awareness and contingency
- S) Synthesis by the receiver

Two particularities of this tool are the anticipation of potential problems (1rst S), and the check-back from the hand-off receiver (2nd S) to ensure the information has been correctly understood. Besides allowing for verification, this check-back also provides an opportunity to clarify certain concepts if needed [11].

The benefits of I-PASS, along with physician training, supervision and process change, have been demonstrated in several studies, including a multicenter study that estimated a 23% reduction in the risk of medical errors and a 30% reduction in the risk of preventable adverse events [2, 10].

Despite an increased awareness about hand-offs and risks for patient safety, a lack of standardization of hand-offs can still be observed in many hospitals [12]. Furthermore, since most hand-off studies are observational, it is difficult to compare the hand-offs between care-providers since there are no two identical clinical situations. Our study design was a simulation study, which put all participants in the same conditions and thus allows the comparison of their medical hand-offs. It reproduced the Friday evening day-to-night hand-off of our institutional division of general internal medicine. The detailed description of the study and the error analysis results are available in a prior publication [13]. Several synonyms are used to describe the hand-off process: for clarity in this paper, we will use the term hand-off to designate when a person *receives* information, and the sign-out for when a person *gives* information.

In this secondary analysis of the hand-off data, we seek to first study the relevance and completeness of these communications to answer the following questions.

- Is there an association between these items and the level of training of the participating physician?
- Do relevance and completeness increase with the duration of the sign-out?

We then analyze the structure of these communications using the I-PASS standardization tool to answer the following questions.

- What is the distribution of information between the I-PASS categories?
- Does their sequence follow a general pattern?
- How could the observed variability be explained?

The purpose of this work is to propose ways of improving medical hand-offs that could be introduced in the training of residents.

## Methods

Our analyses are based on a pre-existing database collected during the simulation study [13] described below. They follow a mixed method with a predominantly qualitative approach.

### Simulation study

Thirty general internists (residents and supervisors) from the University Hospitals of Geneva (HUG) were recruited. The study simulated a hand-off between two residents on a Friday evening in the Internal Medicine division of the HUG. More precisely, the simulation consisted of three phases:

- First, the participant received a standardized hand-off of four clinical cases randomly chosen among the 8 available clinical cases (Additional file 1), with the possibility to ask questions.
- Then, the participant was left alone to begin her shift with access to the electronic medical records (EMR). During this time, she received four phone calls from the nurse following a standardized structure, only two of which were about the clinical cases presented during the initial medical hand-off.
- Finally, the scenario ends with the participant having to interrupt her shift for personal reasons and she signs her patients out to a colleague.

All exchanges were recorded and transcribed. The level of experience and the number of years of practice of the participants were recorded. For this study, we mainly focused on the third part of the simulation, namely the sign-out of a total of 6 clinical cases from the participant to her colleague (played by the principal investigator), randomly selected among the 8 possible cases.

In addition, to constitute the gold standard (GS) of the study, a focus group of three experts (senior doctor in internal medicine) established a consensus on the essential elements of each sign-out, with and without nurse calls. This consensus was the result of a discussion between the experts and focused on the *content* rather than the *structure* of the hand-off.

### Coding of transcripts

We coded each clinical feature in the sign-outs, and defined relevance by its presence in the GS dataset. Each sign-out therefore contained both relevant and irrelevant features.

Based on the codes used by the GS, we created a codebook with 8 main groups, identity (I)—principal diagnosis (Dp)—secondary diagnosis (Ds)—differential diagnosis (DD)—symptoms and clinical signs (S)—complementary examinations (Ec)—treatment (TTT)—follow-up (Sv), each divided into several subgroups.

Using Atlas.ti version 8.4.5. software, we followed an iterative coding process and added codes to the codebook as needed. The coding was finalized by discussion and consensus of the three investigators.

We then allocated the final codes to the I-PASS categories [10], defined as follows: the item I (illness severity) included information about the general condition of the patient (stable/instable) as well as about its urgency and order of priority. Data about the case history and background, events leading up to admissions, hospital course and ongoing assessment were part of the category P (patient summary). Moreover, the category A (action) corresponded to the actions that the doctor needed to carry out during the beginning of her shift (including reactions to complications following nursing calls) and to the actions delegated to the colleague for the rest of the night, while the category S (situation awareness and contingency) represented the doctor's anticipations of potential complications or events (e.g. “If the patient develops fever, then…”). We did not include the second “S” (synthesis by the receiver) at this stage (see below), since the study was not designed to conduct this analysis (the colleague receiving the sign-out was an investigator).

### Analyses

In this paper, we defined relevance as all sign-out elements that were present in the GS dataset. We defined completeness as the proportion of elements in a participant’s sign-out, out of all the elements of the GS dataset for a specific clinical case.

We use descriptive analyses to report the rates of relevance and completeness of the sign-outs. We also studied the distribution of the data mean, median and standard deviation), and included the temporal dimension through a linear regression to illustrate the influence of the duration of the sign-outs on their relevance and completeness. Moreover, we conducted a subgroup analysis according to the level of experience (residents and supervisors).

In a second step, we studied the distribution of I-PASS categories by participant and by clinical case. These results are illustrated in the form of histograms and pie charts. The Chi2 statistical test was used to compare participant and GS results.

Qualitative analysis of the sequence of medical sign-outs was performed using the Eventflow [14] software, which provides a visual approach to compare the sequences of I-PASS categories between clinical cases and between participants.

To study the participants' use of the 2nd S of the I-PASS (Synthesis by the receiver), we completed the current analysis with data from the first part of the simulation, i.e., when the participant was receiving the initial standardized hand-off. We investigated whether some participants spontaneously summarized the hand-off they received.

## Results

### Participants

Thirty physicians from the Department of Internal Medicine of the University Hospital of Geneva participated in the simulation study, including 15 residents and 15 supervisors (staff physician or chief residents). The latter had an average of 6.5 years of additional experience. Only 2/30 physicians had received training in medical sign-out, and the majority (24/30) did not know of or use any mnemonic tools.

Each participant signed out the 6 clinical cases encountered at the beginning of the shift (4 patients from the initial hand-off and 2 new patients for which she recieved a call from the nurse nursing call) for an average of 12 min. Two participants differed significantly from the others: Participant P12 chose to present only 3 clinical cases in 6 min and, conversely, participant P17’s sign-out was very detailed and lasted 30.5 min.

### Coding

Following the iterative coding process described above, we grouped the 3715 participants’ quotes into 523 total codes.

### Relevance

**Fig. 1:**
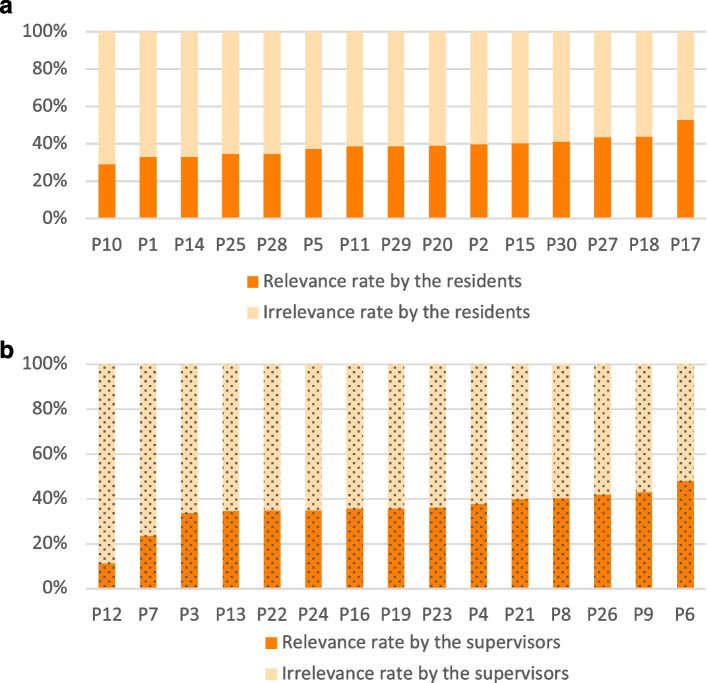
**a** and **b** Relevance rate by the residents and the supervisors. Relevance rates (ordinate, %) by participant (abscissa, one column per participant) in the residents’ group (Fig. 1a, solid fill) and the supervisor group (Fig. 1b, dashed fill), ordered by increasing scores

Relevance ranged from 11.3% to 52.9% among all participants (Fig. 1). In the residents’ group, the mean value was 38.7 ± 7% (median 38.9%), and in the supervisor group, it was 35.6 ± 9% (median 35.8%). These results followed a normal distribution.

**Fig. 2:**
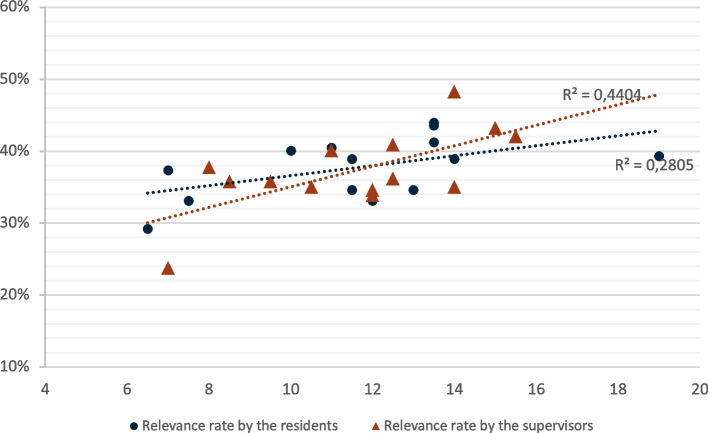
Effects of the hand-off’s duration on its relevance, by level of expertise. *Relevance rate (ordinate, %) by the duration of the hand-off (abscissa, minutes) in the residents’ group (red triangles) and the supervisors’ group (black circles)*

We compared the rate of relevance with the duration of medical sign-out separately for the two groups of residents and supervisors. The linear regression shown in Fig. 2 illustrates a positive correlation between relevance rate and sign-out duration, which was slightly stronger in the residents’ group (R2 = 0.62) compared with the supervisor group (R2 = 0.56).

An analysis without the extreme values (exclusion of P17 and P12 participants) reduced the strength of the association between relevance and sign-out duration, and this even more in the supervisors’ group (R2 = 0.28) compared to the residents’ group (R2 = 0.44) (Additional file 2).

### Completeness

**Fig. 3:**
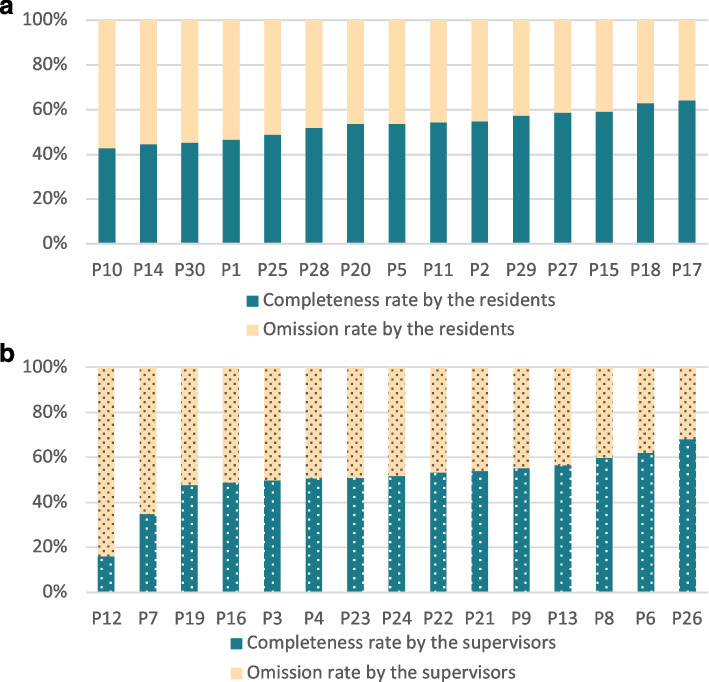
**a** et **b**): Completeness rate by the residents and the supervisors. *Completeness rates (ordinate, %) by participant (abscissa, one column per participant) in the residents’ group (Fig. 3a, solid fill) and the supervisor group (Fig. 3b, dashed fill), ordered by increasing scores*

In the completeness analysis, we obtained an average rate of 53.3 ± 7% (median 53.8%) for the residents’ group and 50.6 ± 12% (median 51.7%) for the supervisor group (Fig. 3). The inter-variability in these results was greater than in the analysis of the relevance: indeed, the participants signed out between 16 and 68% of the information expected from the gold standard.

**Fig. 4:**
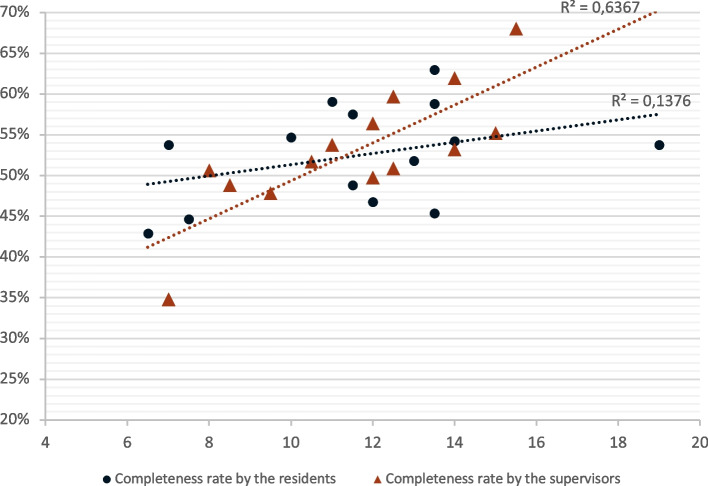
Effects of the hand-off’s duration on its completeness, by level of expertise. *Completeness rate (ordinate, %) by the duration of the hand-off (abscissa, minutes) in the residents’ group (red triangles) and the supervisors’ group (black circles)*

The correlation between completeness rate and sign-out duration, estimated by the Pearson's coefficient (result of a linear regression), was positive for all participants and increased with the level of training (R2 = 0.67 for supervisors and 0.32 for residents) (Fig. 4).

Excluding the extreme values (P17 and P12 participants) had little effect on the correlation coefficient for the supervisor group (R2 = 0.64), but in the residents’ group, it reduced the strength of the association between completeness and duration by a factor of 2.5, although it remained positive (R2 = 0.14) (Additional file 3).

**Fig. 5:**
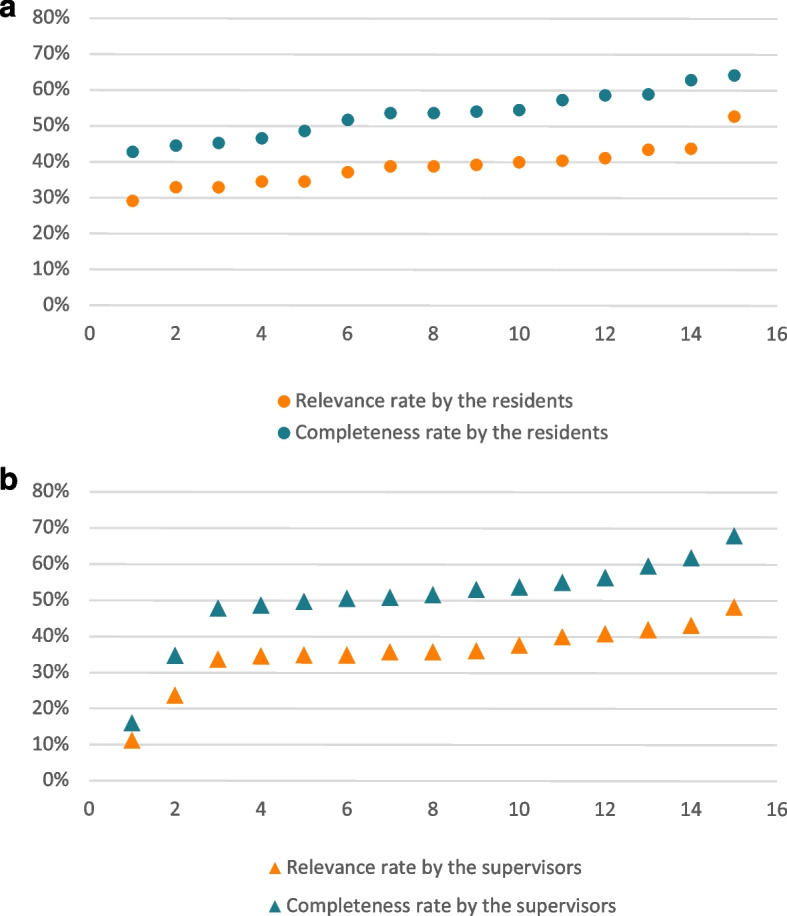
**a** and **b** Correlation between relevance and completeness of hand-offs, by level of expertise. *Relevance (orange, ordinate, %) and completeness (blue, ordinate, %) rates in the residents’ group (Fig. 5a, circles) and the supervisor group (Fig. 5b, triangles) in ascending order of scores*

Combining the results of the relevance and completeness analyses for each participant confirms their strong correlation (R2 = 0.91) (Fig. 5).

### Proportions of I-PAS(S) categories

**Fig. 6:**
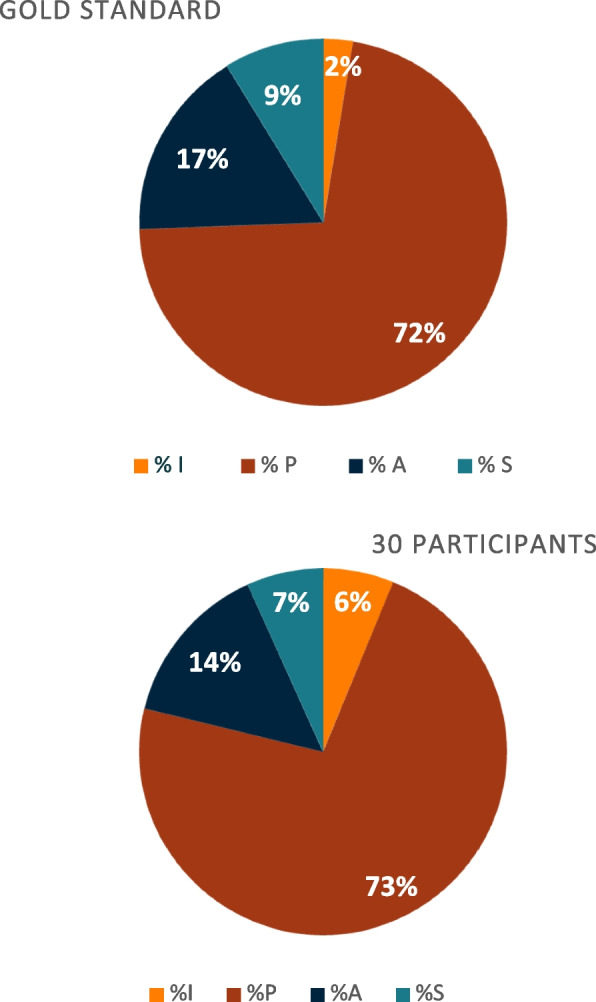
**a** and **b**: I-PAS(S) categories’ distribution in sign-outs, by the gold standard and by the participants. *Distribution of information transmitted according to I-PAS(S) categories (Illness severity, Patient summary, Actions list, Situation awareness). The graph on the left (Fig. 6a) represents the average of the 30 participants and the graph on the right (Fig. 6b) illustrates the distribution within the gold standard sign-out*

Both in the GS and for the participants, the distribution of I-PASS categories across all codes—i.e., the information reported—was highly skewed. Indeed, the proportion of items in category P (patient summary) was much higher than the others, followed by category A (actions to be taken). There were statistically significant differences between the proportions of code included in the categories A (*p* = 0.046) and I (*p* 0.001) for the participants compared to the focus group (Additional file 4), illustrated in Fig. 6.

**Fig. 7:**
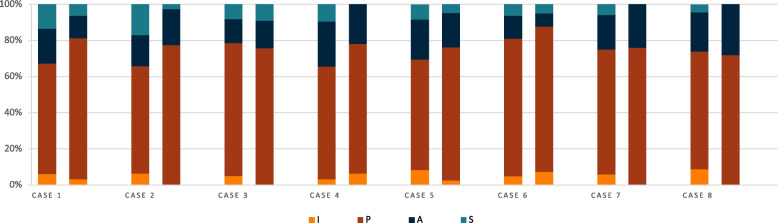
I-PAS(S) categories’ repartition for the eight clinical cases, by the gold standard and by the participants. *Distribution of reported information according to I-PAS(S) categories based on the 8 clinical cases for the average of the 30 participants (left column) and for the gold standard (right column)*

To study the influence of the diversity of the clinical cases on the distribution of the I-PASS categories, represented in the histogram below (Fig. 7), we compared the proportions of each of the 8 clinical cases for the GS and for the participants. For the 30 participants the variations were larger than in the GS and concerned mainly category A (standard deviation = 8.5) and S (standard deviation = 6.5) categories. The results are presented in detail in Additional file 5. For the GS, category I items were absent in clinical cases 2, 3, 7 and 8 and category S items in cases 4, 7 and 8.

### I-PAS(S) category sequences

Most medical sign-outs began with category P (97%), followed by I (2.5%) and A (0.5%). The categories I, A and S most often appeared only in the second part of the sign-out, with category A generally preceding S. The sign-out ended for half of the clinical cases with the category I (52%). The category S, which included anticipations, was absent in a 54% of sign-outs.

When studying the sequences by clinical case, we found that in clinical cases 1 and 5 the four categories are most often represented (72% and 75% respectively). On the contrary, only a few participants shared their anticipation of potential patient complications (category S) for clinical cases 7 (16%), 2 (27%), 6 (31%) and 3 (36%). Additional file 6 summarizes these observations in a table.

When comparing the sequences between participants, it was difficult to find a pattern specific to each physician that was repeated in the sign-out of their 6 clinical cases. Only one participant (P25) began her sign-outs with information about the severity of the disease (category I) for more than one clinical case (3/6). In addition, 5 participants did not communicate any category S information for 5 of their 6 cases. Finally, when comparing the 6 sequences from each participant, there is no generalizable difference in the order of categories between clinical cases that received a nursing call and those that did not. However, we note that there are often more codes from categories A, S, and I in the nurse call cases.

### Receiver's summary of the initial hand-off

During the initial standardized hand-off received by the participant, each of the participants asked questions to clarify information but none systematically summarized the information received to ensure that it was properly understood (2nd S of the I-PASS). Indeed, only one participant (P1) did this for only one of the clinical cases received (case 1).

## Discussion

Analysis of the data collected revealed significant structural differences between spontaneous medical sign-out, the gold standard, and the I-PASS tool.

Firstly, the relevance and completeness rates were highly correlated (R^2^ = 0.91) but relatively low, averaging 37.2% and 51.9%, respectively. This indicates that more than 60% of the information transmitted by the physicians was deemed unnecessary by experts, and almost half of the essential information was missing. This discrepancy can partly be explained by the consensus-based approach to determining relevance and completeness, which is subject to variability in expert opinions. For instance, a study by Charlin showed a 59% difference between group consensus and individual expert responses [15]. Faced with the same clinical situation, the ways of thinking may vary from one clinician to another [16, 17] and diagnostic accuracy is influenced more by thinking about diagnostic hypotheses than by a precise list of information to be sought [18]. Moreover, one study [19] showed that the most important element of a clinical case is not transmitted in 60% of cases, although the transmitting physician is convinced that it has been correctly understood. Communication studies show that speakers often overestimate their effectiveness, leading to omissions and potential errors [20, 21].

Furthermore, the completeness rate did not improve among supervisors, suggesting that experienced physicians might convey information more implicitly [22]. There is currently no teaching about medical communication in the residency, so it is not surprising that it does not seem to improve with professional experience.

As expected, the longer the sign-out, the more likely it was to include all essential elements, but the goal is efficiency, not duration. Optimal hand-offs require selecting relevant information concisely, balancing detail with the risk of misunderstandings [23, 24]. Using mnemonic tools like I-PASS can improve quality by preventing omissions and facilitating comprehension [10]. The second "S" in I-PASS, involving a synthesis by the receiver, can correct misunderstandings and clarify elements.

We observed an uneven distribution of the I-PASS categories both in the gold standard and among participants. Category P includes many elements essential for efficient sign-out, such as patient's personal data, clinical history, reason for hospitalization, potential diagnoses, current clinical situation, and stages of management. The other categories, although equally important, contain fewer elements. This uneven distribution is intentional to highlight the need to emphasize communicating about the patient's severity (I), next steps in management (A), and anticipation of potential complications (S), as these are often omitted during hand-offs [11].

The general pattern of spontaneous sign-outs followed the order P-A-S-I when all categories were represented, with variations in the latter part (A-S-I). About half ended with category I, indicating patient priority, although I-PASS suggests starting with this to convey severity immediately [25]. Only one participant followed this approach consistently.

Comparing the distribution of I-PASS categories among the eight clinical cases within the gold standard, we found that category S was missing in three cases (4, 7, and 8). For instance, in case 4 (retrosternal pain in a heart failure patient), the gold standard suggested going directly to the patient without anticipating further actions. Some participants, however, recommended calling the cardiology department or advancing the planned coronary angiography. In case 7 (fever in a hyponatremic patient), neither the gold standard nor most participants shared recommendations for potential complications, considering the patient stable with necessary examinations already ordered. For case 8 (medication error), the gold standard judged close monitoring sufficient, while over one-third of participants suggested transferring the patient to the intermediate care unit if their condition worsened. Sign-outs involving a nurse call had more A, S, and I codes, indicating acute complications. Omission of urgency, to-do lists, and anticipation in cases without complications highlights the importance of including this information to avoid errors.

Category I was also absent in several clinical cases (2, 3, 7, and 8) within the gold standard, where no information on the severity of the clinical case was provided. Given that these elements improve communication between caregivers [25–27]and are likely not standard practice in the HUG internal medicine department, it would be beneficial to introduce them into internist training explicitly. Conversely, participants included a higher proportion of items in category I. This difference might be because the focus group defined the gold standard items during a shared discussion without following the full on-call simulation scenario. For example, the prioritization of different patients, part of category I, was not explicitly present in the gold standard, whereas, during the simulation, the investigator asked participants about patient prioritization.

The comparison between the gold standard and the sign-outs of the 30 participants revealed a significant underrepresentation of category A among participants. They provided less relevant information on the next steps in management than expected. For example, in case 3, none of the participants thought to stop ibuprofen for a skin rash suspected to be allergic. In case 8 (a patient receiving both a beta-blocker and anti-diabetic medication by mistake), only one participant suggested suspending the next dose of the beta-blocker.

Category S (anticipation of future problems) varied significantly based on the clinical case. For instance, in case 7 (fever in a hyponatremic patient), 84% of participants did not suggest potential complications due to existing actions and the complexity of hyponatremia. Anticipations were more common in cases with prior complications (e.g., case 5) or perceived instability (e.g., case 1).

Regarding the 2nd S of the I-PASS tool (receiver synthesis), the study was not designed to specifically study this, as the receiver was part of the investigation team. However, based on the initial standardized hand-off, only one participant rephrased the received hand-off to ensure understanding. This synthesis is not yet a local habit and should be actively promoted in training.

## Conclusions

### Strengths and limitations

Studying medical hand-offs, particularly oral ones, is challenging due to their dependence on the clinical situation at a given time. To address this, we conducted a simulation study exposing 30 participants to the same standardized clinical scenarios, allowing for comparison of various sign-out elements. Each participant confirmed the realism of these situations, akin to their daily practice. However, the possibility of a Hawthorne effect influencing the results cannot be excluded.

To limit selection bias, each participant managed six clinical cases, sufficient to simulate the realistic conditions of a night shift with adequate complexity. Our gold standard was defined by a consensus of senior doctors, chosen for their expertise as recent ex-residents, though an absolute reference for the ideal hand-off content is difficult to establish. Despite a relatively large sample size of 30 physicians and 180 hand-offs, the data is a few years old and based on a qualitative approach, limiting the generalizability of our findings to the current healthcare landscape.

This study is unique in investigating the sequence of I-PASS categories and comparing them to their content. Additionally, while I-PASS has mainly been studied in the USA, examining its implementation in Switzerland can provide valuable insights through further qualitative studies.

## Conclusion

Through this work, we observed that there are several gaps between the current medical sign-outs in the general internal medicine division of our institution compared to the I-PASS structure, whose effectiveness has been demonstrated. Thus, we can recommend an approach to implement the I-PASS mnemonic with a particular emphasis on the category I to start the sign-out and on the category S to better anticipate and thus prevent complications. Additionally, the implementation of the 2nd S (synthesis by the receiver) should make possible to optimize the flow of the relevant information as previous studies have shown [27–29] without making them longer. Future studies will be needed to assess the impact of our recommendation and adapt the key elements of each letter from the mnemotechnic tool according to the context. Finally, we underline the opportunity to introduce specific hand-off training to senior medical students to optimize the selection of relevant information, to use a structured discourse and to confirm the information received.

## Supplementary Information

Supplementary Material 1.

Supplementary Material 2.

Supplementary Material 3.

Supplementary Material 4.

Supplementary Material 5.

Supplementary Material 6.

## Data Availability

The datasets generated and/or analyzed during the current study are not publicly available due privacy and confidentiality reasons but are available from the corresponding author on reasonable request.

## Abbreviations

GS: Gold standard
HUG: University Hospitals of Geneva
EMR: Electronic medical records
P1-30: Participant 1 to 30
