# Supplementary material for: Secondary analysis of hand-offs in internal medicine using the I-PASS mnemonic
Source: BMC Med Educ. 2024 Sep 27;24:1046. doi: 10.1186/s12909-024-05880-7 (PMC11430516; doi:10.1186/s12909-024-05880-7)
Supplement: Supplementary file 1 — Supplementary Material 1. [file 12909_2024_5880_MOESM1_ESM.docx]

**Additional file 1:** 8 clinical cases from the simulation study.

| **Clinical case** | **Main diagnosis** | **Nurse call** |
| --- | --- | --- |
| **Case 1** | Decompensation of Child C cirrhosis in a patient with chronic alcoholism due to sepsis in a context of upper gastrointestinal bleeding. Hepatic failure with encephalopathy. Recently transferred from continuing care. Currently still delirious. Awaiting paracentesis results for recurrent febrile state. | Hypotension |
| **Case 2** | Acute renal failure with electrolyte imbalance in a patient with ileostomy and COPD. | Dyspnea |
| **Case 3** | Pneumonia with empyema in a young smoker with chest drain pain. | Skin rash |
| **Case 4** | Inaugural cardiac decompensation following cardiac ischemia. Diabetes with systemic complications. Perforated gastric ulcer. | Thoracic pain |
| **Case 5** | Pneumonia in a patient with metastatic prostate cancer with episodes of desaturation. Atelectasis of undetermined origin. OSA. | Thoracic pain |
| **Case 6** | Hepatic decompensation in a patient with cirrhosis stage Child C. Episode of hemoptysis on admission because patient anticoagulated for a recent pulmonary embolism. | Abdominal pain |
| **Case 7** | Incidental discovery of hyponatremia in a patient with suspected cirrhosis. | Delirium with fever |
| **Case 8** | Urosepsis in hypertensive patient. Repeated falls and currently fractured right wrist. | Medication error |
