# Supplementary material for: Secondary analysis of hand-offs in internal medicine using the I-PASS mnemonic
Source: BMC Med Educ. 2024 Sep 27;24:1046. doi: 10.1186/s12909-024-05880-7 (PMC11430516; doi:10.1186/s12909-024-05880-7)
Supplement: Supplementary file 2 — Supplementary Material 2. [file 12909_2024_5880_MOESM2_ESM.docx]

**Additional file 2:** Relevance rate by transmission time without extreme values (P12 and P17).


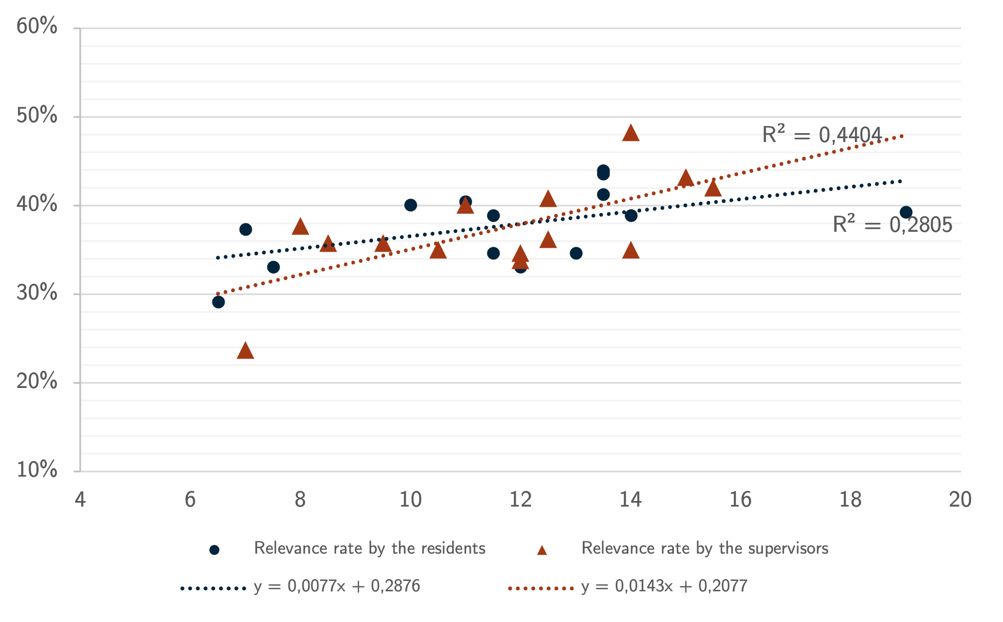


Figure 9: *Relevance rate (%) by transmission time (minutes) for the residents’ group (red triangles) and for the supervisors’ group (black circles) without extreme values.*
