# Supplementary material for: Secondary analysis of hand-offs in internal medicine using the I-PASS mnemonic
Source: BMC Med Educ. 2024 Sep 27;24:1046. doi: 10.1186/s12909-024-05880-7 (PMC11430516; doi:10.1186/s12909-024-05880-7)
Supplement: Supplementary file 4 — Supplementary Material 4. [file 12909_2024_5880_MOESM4_ESM.docx]

**Additional file 4:** Mean and median in the distribution of IPAS(S) categories by the 30 participants. Since the median and mean are very close, it is possible to use the mean of all 30 participants in a statistically significant way.

|  | **Mean by the 30 participants** | **Median by the 30 participants** | **Difference (Mean – median)** |
| --- | --- | --- | --- |
| **I** | 6,2 | 6,4 | 0,2 |
| **P** | 73,3 | 72,7 | 0,6 |
| **A** | 13,8 | 14,8 | 1,0 |
| **S** | 6,7 | 6,5 | 0,2 |


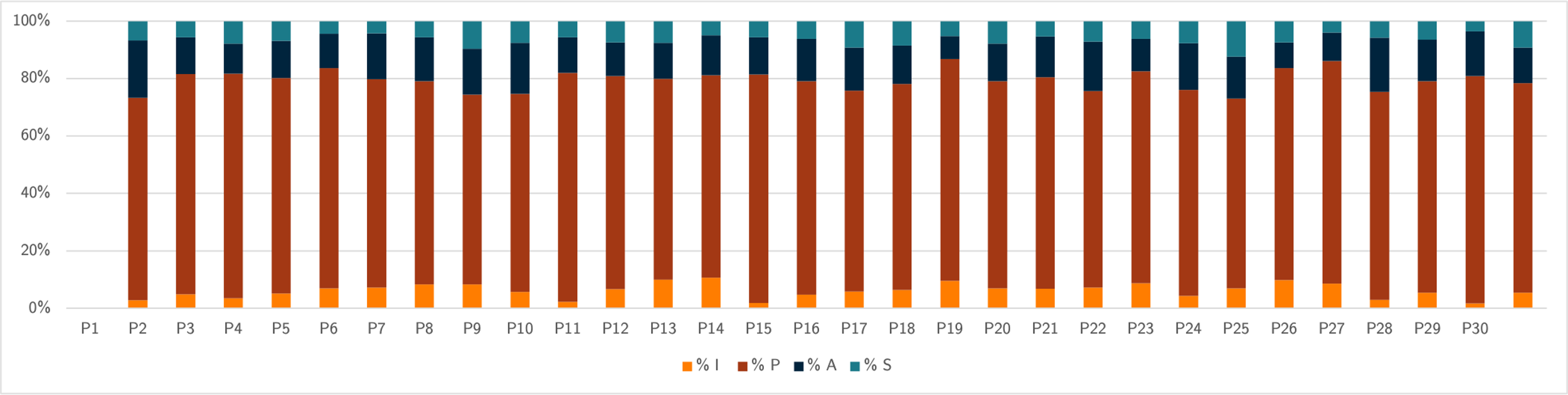


Figure 11: *Proportions of IPAS(S) categories (%) per participant (30).*
