# Supplementary material for: Secondary analysis of hand-offs in internal medicine using the I-PASS mnemonic
Source: BMC Med Educ. 2024 Sep 27;24:1046. doi: 10.1186/s12909-024-05880-7 (PMC11430516; doi:10.1186/s12909-024-05880-7)
Supplement: Supplementary file 5 — Supplementary Material 5. [file 12909_2024_5880_MOESM5_ESM.docx]

**Additional file 5:** IPAS(S) categories distribution

Variance and standard deviation in the distribution of IPAS(S) categories among clinical cases.

|  | **Standard deviation by the gold standard** | **Standard deviation by the 30 participants** |
| --- | --- | --- |
| **I** | 2,99 | 6,48 |
| **P** | 3,02 | 3,36 |
| **A** | 6,64 | 8,46 |
| **S** | 3,38 | 5,42 |

Table of results of IPAS(S) category distributions by participant for the average of the 30 participants (left column) and for the gold standard (right column).

| Clinical cases | Mean by the 30 participants | Gold standard |
| --- | --- | --- |
| 1 | I=6,0 P= 61,2 A=19,4 S=13,4 | I=3,1 P= 78,1 A=12,5 S=6,3 |
| 2 | I=6,3 P= 59,4 A=17,2 S=17,2 | I=0 P= 77,5 A=20,0 S=2,5 |
| 3 | I=4,9 P= 73,8 A=13,1 S=8,2 | I=0 P= 75,7 A=15,2 S=9,1 |
| 4 | I=3,1 P= 62,5 A=25,0 S=9,4 | I=6,3 P= 71,8 A=21,9 S=0 |
| 5 | I=8,3 P= 61,1 A=22,2 S=8,3 | I=2,4 P= 73,8 A=19,0 S=4,8 |
| 6 | I=4,8 P= 76,2 A=12,7 S=6,3 | I=7,3 P= 80,5 A=7,3 S=4,9 |
| 7 | I=5,8 P= 69,2 A=19,2 S=5,8 | I=0 P= 75,9 A=24,1 S=0 |
| 8 | I=8,7 P= 65,2 A=21,7 S=4,3 | I=0 P= 72,0 A=28,0 S=0 |
