# Supplementary material for: Secondary analysis of hand-offs in internal medicine using the I-PASS mnemonic
Source: BMC Med Educ. 2024 Sep 27;24:1046. doi: 10.1186/s12909-024-05880-7 (PMC11430516; doi:10.1186/s12909-024-05880-7)
Supplement: Supplementary file 6 — Supplementary Material 6. [file 12909_2024_5880_MOESM6_ESM.docx]

**Additional file 6:** Table summarizing observations from the graphical analysis of IPASS category sequences using Eventflow software.

| Clinical cases | 4 categories present (I-P-A-S) | Elements from category S | Elements from category I | Only elements from categories P and A |
| --- | --- | --- | --- | --- |
| 1 | 72,00% | 80,00% | 80,00% | 4,00% |
| 2 | 22,73% | 27,27% | 81,82% | 4,55% |
| 3 | 22,73% | 36,36% | 45,45% | 40,91% |
| 4 | 50,00% | 60,00% | 85,00% | 5,00% |
| 5 | 78,26% | 86,96% | 91,30% | 0,00% |
| 6 | 23,08% | 30,77% | 65,38% | 34,62% |
| 7 | 15,79% | 15,79% | 78,95% | 21,05% |
| 8 | 31,58% | 42,11% | 63,16% | 26,32% |
